# Supplementary material for: German Version of the Child Sexual Abuse Myth Scale (CSAMS-G): Translation, Expansion, and Construct Validation
Source: Behav Sci (Basel). 2025 Jan 28;15(2):143. doi: 10.3390/bs15020143 (PMC11851446; doi:10.3390/bs15020143)
Supplement: Supplementary file 1 [file behavsci-15-00143-s001.zip › CSAMSG_supplementary_material.pdf]

Supplementary material

German version of the Child Sexual Abuse Myth Scale (CSAMS-G) – Translation, Expansion, and Construct Validation

S1: Measurements

1. GHQ-12 (General Health Questionnaire; a questionnaire to assess the mental and physical health of participants)
2. CE-CSA (Cognitions and Emotions about Child Sexual Abuse [*30*]; this measurement checking the attitudes of the participants towards CSA on a cognitive and emotional level)
3. TSiPo (Trust Scale in Police; a questionnaire to examine the participants' trust in law enforcement)
4. ETRAS-CSA (the German and expanded version of the TRAS-CSA by Walsh et al. [*31*] to analyze the attitudes towards reporting CSA)
5. WewL (Values and value-orientated living; a questionnaire to assess the values of the participants)
6. CSAMS-G

S2: German version of the CSAMS-G

Im folgenden Fragebogen werden Ihnen Aussagen zum Thema des sexuellen Kindesmissbrauchs gezeigt. Bitte geben Sie zu jeder Aussage an, inwieweit Sie dieser zustimmen („Lehne ab" bis „Stimme zu"). Bitte beantworten Sie die Aussagen möglichst spontan. Wenn Sie sich unsicher sind, wählen Sie die Antwort aus, der Sie am ehesten zustimmen.

		**Lehne ab**	**Lehne eher ab**	**Stimme eher zu**	**Stimme zu**
**1**	Sexueller Kontakt zwischen einem Erwachsenen und einem Kind, den das Kind will und der für das Kind körperlich angenehm ist, kann im Grunde nicht als „missbräuchlich" bezeichnet werden.	☐	☐	☐	☐

**2**	Sexuelle Kontakte mit einem Erwachsenen können sich positiv auf die weitere psychosexuelle Entwicklung des Kindes auswirken.	☐	☐	☐	☐
**3**	Die meisten Kinder werden von Fremden oder Personen, die sie nicht gut kennen, sexuell missbraucht.	☐	☐	☐	☐
**4**	Kinder, die sich verführerisch verhalten, sind zumindest teilweise für die sexuelle Reaktion des Erwachsenen verantwortlich.	☐	☐	☐	☐
**5**	Sexueller Kontakt zwischen einem Kind und einem Erwachsenen, der ohne Gewalt oder Zwang verläuft und bei dem es nicht zu versuchtem oder stattgefundenem Geschlechtsverkehr kommt, wird wahrscheinlich keine schwerwiegenden psychischen Folgen für das Kind haben.	☐	☐	☐	☐
**6**	Eine Frau, die ihren Partner sexuell nicht befriedigt, ist teilweise verantwortlich, wenn sich ihr Partner aus Frustration sexuelle Befriedigung bei ihren Kindern sucht.	☐	☐	☐	☐
**7**	Sexueller Missbrauch an Kindern kommt vor allem innerhalb von Familien vor, die in Armut und ungeordneten, instabilen Verhältnissen leben.	☐	☐	☐	☐
**8**	Nicht der sexuelle Kontakt mit Erwachsenen ist für Kinder schädlich, sondern die soziale Stigmatisierung, die entsteht, wenn das „Geheimnis“ bekannt wird.	☐	☐	☐	☐
**9**	Viele Kinder haben den unbewussten Wunsch nach einer sexuellen Beziehung zu einem andersgeschlechtlichen Elternteil und begünstigen deshalb mit ihrem Verhalten unbewusst einen sexuellen Missbrauch durch diesen Elternteil.	☐	☐	☐	☐
**10**	Heranwachsende Mädchen, die sehr freizügige Kleidung tragen, fordern den sexuellen Missbrauch heraus.	☐	☐	☐	☐

**11**	Kinder von schwulen oder lesbischen Paaren haben ein größeres Risiko sexuell missbraucht zu werden als Kinder heterosexueller Paare.	☐	☐	☐	☐
**12**	Jungen genießen sexuellen Kontakt mit Erwachsenen eher als Mädchen und haben daher ein geringeres Risiko, durch die Erfahrung traumatisiert zu werden.	☐	☐	☐	☐
**13**	Sexueller Kindesmissbrauch wird durch gesellschaftliche Probleme wie Arbeitslosigkeit, Armut und Alkoholmissbrauch verursacht.	☐	☐	☐	☐
**14**	Wenn Kinder andauernden sexuellen Missbrauch nicht melden, wollen sie, dass der sexuelle Kontakt fortgesetzt wird.	☐	☐	☐	☐
**15**	Ältere Kinder mit einem besseren Verständnis von Sexualität sind selbst dafür verantwortlich, sich gegen sexuelle Annäherungsversuche durch Erwachsene zu wehren.	☐	☐	☐	☐
**16**	Kinder, die sexuellen Missbrauch erlebt haben, können ihre Missbrauchserfahrungen sprachlich nicht ausdrücken.	☐	☐	☐	☐
**17**	Sexueller Kindesmissbrauch geht von einem bestimmten Täter:innentypus aus.	☐	☐	☐	☐
**18**	Sexueller Kindesmissbrauch findet unter der Anwendung von Gewalt statt, welche eindeutige physische Spuren hinterlässt.	☐	☐	☐	☐
**19**	Es gibt Verhaltensweisen von Kindern, die eindeutig dafür sprechen, dass sexueller Missbrauch stattgefunden hat.	☐	☐	☐	☐

Verharmlosung des sexuellen Kindesmissbrauchs: Items 1, 2, 5, 8, 9, 12, 14; Verschiebung der Verantwortung: Items 4, 6, 10, 15; Annahmen über Täter:innen: Items 3, 7, 11, 13, 17; Falsche Überzeugungen bezüglich sexuellen Kindesmissbrauchs: Items 16, 18 ,19.
